# Ambivalence related to potential lifestyle changes following preventive cardiovascular consultations in general practice: A qualitative study

**DOI:** 10.1186/1471-2296-9-50

**Published:** 2008-09-12

**Authors:** Dea Kehler, Bo Christensen, Torsten Lauritzen, Morten Bondo Christensen, Adrian Edwards, Mette Bech Risør

**Affiliations:** 1 The Research Unit and Department of General Practice, Institute of Public Health, University of Aarhus, Vennelyst Boulevard 6, 8000 Aarhus C, Denmark; 2 Department of General Practice, Institute of Public Health, University of Aarhus, Vennelyst Boulevard 6, 8000 Aarhus C, Denmark; 3 Research Unit of General Practice, Institute of Public health, University of Aarhus, Vennelyst Boulevard 6, 8000 Aarhus C, Denmark; 4 Department of Primary Care and Public Health, School of Medicine, Cardiff University, Heath Park, Cardiff CF14 4XN, Wales, UK; 5 The Research Clinic for Functional Disorders and Psychosomatics, Aarhus University Hospital, Barthsgade 5, 1. 8200 Aarhus N, Denmark

## Abstract

**Background:**

Motivational interviewing approaches are currently recommended in primary prevention and treatment of cardiovascular disease (CVD) in general practice in Denmark, based on an empirical and multidisciplinary body of scientific knowledge about the importance of motivation for successful lifestyle change among patients at risk of lifestyle related diseases. This study aimed to explore and describe motivational aspects related to potential lifestyle changes among patients at increased risk of CVD following preventive consultations in general practice.

**Methods:**

Individual interviews with 12 patients at increased risk of CVD within 2 weeks after the consultation. Grounded theory was used in the analysis.

**Results:**

Ambivalence related to potential lifestyle changes was the core motivational aspect in the interviews, even though the patients rarely verbalised this experience during the consultations. The patients experienced ambivalence in the form of conflicting feelings about lifestyle change. Analysis showed that these feelings interacted with their reflections in a concurrent process. Analysis generated a typology of five different ambivalence sub-types: perception, demand, information, priority and treatment ambivalence.

**Conclusion:**

Ambivalence was a common experience in relation to motivation among patients at increased risk of CVD. Five different ambivalence sub-types were found, which clinicians may use to explore and resolve ambivalence in trying to aid patients to adopt lifestyle changes. Future research is needed to explore whether motivational interviewing and other cognitive approaches can be enhanced by exploring ambivalence in more depth, to ensure that lifestyle changes are made and sustained. Further studies with a wider range of patient characteristics are required to investigate the generalisability of the results.

## Background

Psychological [1-7], sociological [8-11], medical [12-23], educational [24,25] and anthropological [26,27] traditions have all contributed extensively to our knowledge about lifestyle change processes. They have identified key psychological constructs such as different kinds of resistance mechanisms related to motivation and lifestyle change [28-30]. Since 1999, the Danish College of General Practitioners has dedicated 'preventive consultations' comprising motivational interviewing (MI) [7,31] as an approach in the prevention of lifestyle related diseases such as CVD. The preventive consultation is a scheduled consultation focusing on individual prevention and risk reduction strategies, where the person is aware of the agenda such as diet, physical activity, smoking, alcohol or other issues in advance and therefore able to prepare himself for the consultation. An agreement is sought about treatment goals to meet public health priorities towards decreasing the risk of: diabetes, CVD, cancer, osteoporosis, chronic obstructive lung disease, asthma, chronic muscle diseases and mental health conditions [31]. MI is a client-centred and supportive counselling method aimed at enhancing readiness for change by eliciting the client's own motivations for change [7]. The evidence base for MI is strong in the areas of addictive and health behaviour and in research settings MI has out-performed "traditional advice giving" in the treatment of a broad range of behavioural problems and diseases [12]. Furthermore, meta-analysis has shown a significant and clinically relevant effect for MI on combined risk factor profiles including body mass index, total cholesterol, blood pressure, alcohol consumption and a significant, in approximately three out of every four studies. [12] Forty-six out of 72 trials also showed benefits on individual lifestyle risk factors with most success for obesity and alcohol consumption, and less effect on smoking [12]. Improvements were more likely, when more than one encounter had taken place, but further studies were recommended to examine the implementation and effectiveness of MI in daily clinical work [12]. However, little is known about the motivational content in preventive cardiovascular consultations in general practice or their general effectiveness in achieving lifestyle change and risk reduction.

The present study is inspired by current recommendations to use MI in primary preventive strategies and the empirical and multidisciplinary body of scientific knowledge on the importance of motivational aspects regarding potential lifestyle changes. The aim of the study was to explore and describe motivational aspects related to potential lifestyle changes in preventive consultations in general practice among patients at increased risk of CVD. This study presents ambivalence and a typology of subtypes as the core motivational aspect among patients at increased risk of CVD following preventive consultations with their GPs. These GPs had not been specifically educated or trained in MI but introduced to MI through guidelines by the Danish College of General Practitioners on how to use MI in clinical practice.

## Methods

This qualitative study draws its data from 12 one-to-one interviews conducted within 2 weeks after preventive consultations, which were videotaped. In all 30 GPs were included from the Health Insurance Register in Vejle and Aarhus Counties and sampled purposefully in relation to age, gender, communicative education and preventive consultation activity. This helped us to ensure that the sample reflected the range of GPs involved in the daily care of patients at increased risk of CVD on the basis of their preventive service experience and the public guidelines. Seven female and five male GPs mostly from group practices participated. The GPs had worked as practitioners for an average of 12.8 years and their average age was 47.7 years. Three of 12 GPs had prior education and training in MI, another three had psychological training from Balint groups and one from a cognitive therapy course.

The 12 participating GPs recruited 12 patients purposefully in accordance with the risk criteria: 20% or higher risk of contracting CVD within the next 10 years, assessed by the risk score system Precard [32] and variability in the person criteria: gender, age and education selected to address the purpose of the study and to gather information rich data. PRECARD is the model for the Heart Score software, developed by the European Society of Cardiology, which aims to provide detailed risk assessment tools in the area of cardiovascular disease to European countries in general [32]. Purposeful sampling implies that participant and other data sources are chosen, because of their importance to the purpose of the study [33].

Table 1 shows the patients' characteristics and summarises their actual risk factors that were being addressed in the consultations. Two women and 10 men participated; their average age was 57.8 years and they came from different social classes and had attained varying educational levels.

**Table 1:** Characteristics of the patients

Informants	Gender	Age	Employment	Risk factors	Co-morbidity
1	♂	74	Factory worker and pensioner	Hypertension, Hypercholesterolemia, Ex-smoker and Overweight	None
2	♂	69	Taxi owner and pensioner	Hypertension, Smoker, Hypercholesterolemia and IGT*	None
3	♀	57	Factory worker, early retired	Hypertension, Hypercholesterolemia Smoker, Overweight and IGT*	Fibromyalgia
4	♂	43	Drilling platform worker	Smoker, Hypercholesterolemia and Overweight	Knee problems
5	♂	73	Engineer and pensioner	Hypertension, Hypercholesterolemia and Ex-smoker	None
6	♂	51	Interpreter	Hypertension, Hypercholesterolemia and Smoker	None
7	♀	42	Architect and manager of the firm	Hypercholesterolemia, Overweight and Hypertension	None
8	♂	54	Manager in the provision industry	Hypercholesterolemia, Hypertension, Smoker, Overweight and IGT*	None
9	♂	49	Gardener	Hypercholesterolemia, Hypertension, Overweight and Ex-smoker	None
10	♂	69	Grocer and pensioner	Hypercholesterolemia, Hypertension and Smoker	None
11	♂	48	Manager and municipal politician	Hypercholesterolemia, Hypertension, Smoker and overweight	None
12	♂	65	Dock worker and pensioner	Hypercholesterolemia, Hypertension and Overweight	None

* Insulin glucose intolerance

A pilot interview study (n = 3) was conducted during the development of the interview guide focused on motivation [Table 2], which was continually modified as new themes emerged from the data. The first author conducted the one-hour interviews within 2 weeks after the consultation, because we aimed to describe motivational aspects of lifestyle change immediately after the consultation and to reduce recall bias related to memory of the consultation at later stages. Before each interview the research interviewer saw the videotaped consultation belonging to the specific person (mean duration 18 minutes) in order to inform and 'qualify' the interview guide, i.e. to ensure that the subsequent interview with the patient addressed issues relevant to his/her specific consultation. For instance when interviewing about influential motivational factors, the videotaped consultations were used to qualify and focus the interview guide on the specific risk factors considered in the consultation. Consequently the videotaped consultation was not used as a primary data source. The patients did not see the video before the interview. During the interviews, the patients were first asked to recall whatever they remembered from the consultation and how they felt about it. They were then encouraged to describe their experiences and to explore, in particular, their readiness to change lifestyle. They were prompted to address five groups of questions addressing motivational aspects shown in table 2. The interviews were transcribed verbatim by a trained secretary and the first author, read and coded independently and discussed after coding of each interview with the last author of the article, an experienced qualitative researcher.

**Table 2:** Motivational questions in the interview guide

Questions to patients	
Introductive	How do you understand the word motivation?
	Describe what you remember from the consultation about motivation and how you felt about it?
	
Question 1	Did you feel ready to accept your GP's preventive consultation offer?
	
Question 2	How ready were you to change lifestyle before and after the consultation?
	a. Tell me about your readiness to change lifestyle before the consultation?
	b. Tell me about your readiness to change lifestyle after the consultation?
	c. If you were not ready to change lifestyle, then tell me why?
	
Question 3	Which aspects influence your motivation to change lifestyle in general and in the consultation?
	a. Which aspects or factors make you ready to change lifestyle?
	b. Which aspects or factors inhibit your readiness to change lifestyle?
	
Question 4	Describe how your GP tried to motivate you in the consultation and what you felt about it?
	
Question 5	Which persons or networks have the greatest influence on your readiness to change lifestyle?

### Analysis

Coding of themes followed the objectivistic rules of Grounded Theory [33,34] and was carried out through four phases supported by the software program N-vivo 2.0. In the *open coding phase*, the 'motivational phenomena' present in the interview quotations were coded and divided into categories and sub-categories. In the *axial coding phase*, each category was analysed in order to identify its dimensions and characteristics. In the *selective coding phase*, the main categories were generated and named, given their empiric contents. Finally the ambivalence sub-types were generated and described under the overall concept of ambivalence, which was identified as the core motivational aspect (for further details see Table 3). Throughout these four phases the first author (DK) carried out the initial coding of the interviews. Subsequently, the last author (MBR) also read and identified all codes from the interviews then meeting to discuss, add or revised the coding and analytical core categories of each interview by consensus.

**Table 3:** Analytical coding phases in the conceptualisation of ambivalence and the different subtypes.

Grounded Theory Coding phases	Descriptions	Categories
The open coding phase	Identified categories and their antagonistic relations	To be at high risk of cardiovascular disease, having cardiovascular disease, to be healthy, to be unhealthy, to know about illness, to know enough, to change life style, to live unchanged, to take medicine, to live without medicine, to add risk, not to add risk, preventive demands from the health care system, the GP or the family, preventive demands from the risk patient themselves, to contain risk, to act preventively, to know about risk, a lifestyle with stress and many demands, a lifestyle without stress and fewer demands, priority of free time and/or health and/or physical activity and/or family and/or resources

The axial coding phase	The common dimension of the categories and their characteristics	Conflicting feelings and reflections regarding:
		1. To have cardiovascular disease versus to be at high risk of cardiovascular disease. 2. To be healthy versus to be unhealthy. 3. To know about risk/disease versus not to know. 4. To know about risk/disease versus to know enough. 5. To change lifestyle versus to live on without changes. 6. To take medicine versus to live without medicine. 7. To change lifestyle versus to take medicine. 8. To add risk to life versus to stay status quo. 9. Preventive demands from the health care system versus preventive demands from the risk patient. 10. Preventive demands from the general practitioner versus the demands from the risk patient. 11. Preventive demands from the network such as family versus the risk patient's own demands. 12. To know about risk versus to contain risk. 13. To contain risk versus to decide to do something about risk. 14. To know about risk versus to act preventively. 15. Priority of spare time or family or work or physical activity versus priority of the risk patients' own resources and health in every day life. 16. To live a stressed life with many demands versus to live an unstressed life with fewer demands

The selective coding phase	The antagonistic categories with their two dimensions were collected into main categories and named on behalf of their empirical characteristics leading to the main concept of ambivalence, its different sub-types and the concurrent reflective process	Main category 1: Perception ambivalence (sub-categories 1+2) Main category 2: Demand ambivalence (sub-categories 9–11 and 16). Main category 3: Information ambivalence (sub-categories 3+4, 12+14) Main category 4: Priority ambivalence (sub-category 15). Main category 5: Treatment ambivalence (sub-categories 5–8, 13)

The theory or concept generating phase	Definition and types of ambivalence	Ambivalence was defined by conflicting feelings that were found to interact with patients' reflections on lifestyle changing in an iterative and concurrent process. Our analysis brought forward five different ambivalence sub-types: perception, demand, information, priority and treatment ambivalence.

Data were collected, prepared, analysed and interpreted in a concurrent repetitive process involving the empirical material (researcher and participants constructions of the consultation), inspiring theoretical aspects and the study objectives in line with the constructivist grounded theory approach [34] and the so-called analytical "round dance" [35]. By the term "analytical round dance" is meant that the qualitative process from the data collection phase to the final analytical interpretation of the empiric data process is a dynamic and fluid process where the empiric data interacts with the methodological strategies such as for instance the purposeful sampling and the constant comparative strategy and the relevant theories such as for instance motivational interviewing and other behavioural change theories at each step of the analysis. Given the grounded theory method, the focus of the interviews developed and became more theoretically specific as the sequence of interviews progressed; that is, data capture was driven by emerging categories and theory development [34].

During the conduct and analysis of the 12 interviews, the emerging categories were examined for theoretical saturation [33,34] i.e. to examine whether further comparisons, properties or relationships developed or new theoretical insights were revealed [34] (but not for saturation to achieve representativeness).

## Results

Our analysis identified that ambivalence and different ambivalence subtypes: *perception, demand, information, priority and treatment *ambivalence were the core motivational aspects related to lifestyle change following preventive consultations. The following section will describe these subtypes as the main results of our study and offer quotations to illustrate common themes.

### Perception ambivalence

*Perception ambivalence *captured ambivalent feelings and reflections related to patients' perception of being at risk of CVD, on the one hand, and their perception of being healthy or sick on the other. For instance a 42-year old female found it difficult to separate her perception of being at risk from being healthy or suffering from a CVD:

It is a difficult situation when you are on your way to an unhealthy lifestyle. When is it unhealthy and when is it not? When do you have to stop and prevent heart disease?

How do you separate risk from being healthy and/or suffering from cardiovascular disease? How do I convince myself about the fact that I should act preventively, when I feel well? These conflicting feelings and thoughts fill my head after the consultation. (id 7)

Our analysis of patients' perception ambivalence furthermore showed that the conflicting (ambivalent) feelings seemed to interact with the patients' reflections in a concurrent process. These reflections were person-specific and related to aspects such as knowledge, considerations and actions related to lifestyle change. A 69-year-old male informant said:

You feel stupid, when you consult the doctor and he confirms what you already know and think, and, even so, you are unable to act, because you are filled with conflicting feelings about lifestyle changes. The feelings disturb the thoughts. I know what it is all about – it is just so difficult to get my act together. Actually, to know about risk, health and lifestyle habits is very different from being able to consider ... and to consider risk is not always the same as being able to act preventively. (id 10)

### Demand ambivalence

The *demand ambivalence *was due to conflicting demands from the health care system/the GPs/the family, on the one hand, and the patients' own demands on the other hand. This generated confusion about which demands patients should meet. A 65-year-old male informant said:

My views on risk of cardiovascular disease seem to be different from the demands from the health care system, my family or my doctor ..... If you cheat the doctor by just saying something you don't do, or pretend to do, you are cheating both the system and yourself. My doctor talked about the fact that we have to estimate my risk of dying from heart disease..... For me risk of disease is something I should deal with when I grow old. I am still young and have no symptoms, so why should I comply with these demands? (id 12)

The patients experienced furthermore that the demand ambivalence often gave rise to strong personal feelings of stress, which was rooted in and arose from the many and conflicting demands from working life, family or the patients themselves, which affect their confidence and ability to implement lifestyle change. They explained how their feeling of stress eroded their focus on health, made them less aware of their body and more concerned. They felt caught in a vicious circle with limited control over their own lives; a situation where family, work and other external circumstances exercised more control than they did themselves. The informants described furthermore, how their demand ambivalence and feeling of stress could result in conflicting choices between a stressed life with much result-oriented activity versus an unstressed life with lower activity and subsequently fewer demands to achieve results. A 48-year-old male informant said:

Physically, I became increasingly inactive because of stress, work demands ...... Then concerns about my health increased and so did my weight. Then it was just more difficult for me to be physically active and I became more stressed and more passive about prevention – it became a vicious circle. I experienced being so stressed in periods that I did not listen to the signals from my body and lost my will. Suddenly, I was resistant to lifestyle changes. (id 11)

### Information ambivalence

The *information ambivalence *captured uncertainty about how much information the patients actually preferred and from whom. The patients often used their GPs' information as a starting point immediately after the consultation, even though it made them ambivalent. A 69-year-old male informant said:

I don't really know how much information I need. Too much information could make me confused, too little information could make me unaware that I am at risk. I feel that my doctor's information is important, but it makes me unsure what to do. (id 2)

The information ambivalence also contained conflicting feelings and reflections on the sufficiency of information in the consultation, varying from one situation to another. As a 43-year-old male informant said:

Sometimes a little information about my health is sufficient if that's just what I need. Some other times I feel I need much more information. (id 4)

As well as the amount of information causing tensions, the type of information was also important. For instance, the patients found it difficult to interpret and respond to numbers offered in explanations of risk, concepts and definitions. A 54-year-old male informant said:

I like a combination of approaches such as pictures, colours or figures combined with ordinary words and numbers. Then I feel informed. If my GP uses numbers to communicate complex medical risk concepts, then I don't feel informed in a way, because I cannot respond. Besides, how do I know if I am the one who goes free or the one who gets ill? ...... I just need ordinary words, numbers and visual information to feel informed. (id 8)

### Priority ambivalence

*Priority ambivalence *was due to assigning priority in life in relation to working life, health, family and own life and resources.

Priority ambivalence derived for example from either a low commitment or inclination to prioritise health, or physical barriers such as back or knee pain, preventing the patients from changing their exercise habits. The work, family or social networks strained their resources and were given higher priority than their health. A low health priority was, furthermore, related to a low readiness to change lifestyle than a high health priority. A 49-year-old male informant said:

it is difficult to be physically active. Besides, I often experience that my family life and activities force me to reduce my own health preventive activities and spare time and ..... To change lifestyle is about setting your own targets. ....... Low priority of health goes against a healthy lifestyle. (id 9)

and another 42 year-old female informant:

Every day you have to make priorities and with family and fulltime work it can be difficult to prioritize health in your daily living in practice. If you really want to change lifestyle you must prioritize it as an important agenda in your every day living. (id 7)

### Treatment ambivalence

*Treatment ambivalence *typically consisted of ambivalent feelings and reflections about the need to change one's lifestyle and take medicines. Patients would prefer to adopt one or the other approach, but doctors often characterised it as a "both and" situation among high-risk patients. High-risk patients are typically recommended to change lifestyle and take treatment, but they do not always accept this – i.e. they are placed in an ambivalent position. The added risk of taking medicine refers especially to the risk of suffering from side effects versus changing lifestyle. This was frequently mentioned in the interviews. A 43-year-old male informant said:

It is just much easier to take medicine than to change lifestyle. If I could, I would prefer medical treatment, because lifestyle changes are so difficult. On the other hand, taking medicine also carries a risk of side effects – an added risk. (id 4)

### Common features of the ambivalence sub-types

After a preventive consultation, many patients identified issues that reflected ambivalence, although these had not been raised either by the person or by the GP during the consultation. A 42-year-old female informant said:

I felt alone with these contradictory feelings and thoughts, and my doctor did not go into it. But, of course, if I don't tell him, he doesn't get to know these feelings and reflections. It made me unsure, ..., and it reduced my desire for changing lifestyle. (id 7)

A common feature of the ambivalence sub-types was furthermore that patients seemed to shift between different conflicting feelings and reflections in a concurrent and iterative process. Thus their reflections did not always result from conflicting feelings; it was often the other way around. As a 43-year-old male informant said:

Both before and following the consultation, I was filled with conflicting feelings and multiple thoughts related to lifestyle changing and medical treatment at the same time. (id 4)

## Discussion

### Summary of findings

Ambivalence and its subtypes were the core motivational aspects related to potential lifestyle changes in the interviews, even if they were not verbalised during these consultations. The patients perceived ambivalence as conflicting feelings about lifestyle change. These feelings interacted with their reflections in a concurrent and iterative process, ultimately making them decide whether or not to attempt lifestyle change. The analysis allowed us to generate an ambivalence typology consisting of five sub-types, each reflecting a unique dimension of the overall concept (and problem) of ambivalence: perception, demand, information, priority and treatment ambivalence.

### Strengths and weaknesses of the study

The pilot study was useful for optimizing the interview guide, which was further elaborated and refined as the interviews progressed by focusing on derived analytical categories from preceding interviews. Specific questions were inspired by the videotaped consultation of each informant. The videos were made without the presence of the researcher and were not a primary data source. The patients were sampled purposefully by the GPs on the basis of specific instructions reflecting the guidelines on preventive consultations, the purpose of the study and to gather theoretical rich data. GPs were included through the health insurance register in two counties and sampled purposefully. However, some of the participating GPs may have had certain professional interests in preventive consultations. This may have shaped their choice of patients, so that they would either include rather more straightforward cases, perhaps including patients with higher than average health literacy and interest in health and lifestyle change. There was some evidence of these characteristics in the sample, but also of more problematic cases. The GPs were all aware of the guidelines related to MI but had different preventive consultation activity and competence in regarding to preventive consultations and MI. Three of the GPs had special training in MI, which may have shaped their ability to use MI and consider ambivalence for patients in the consultations. Furthermore, the non systematic use of MI must be perceived as a limitation of the results, although this is likely to reflect the reality of current clinical practice. A systematic use of MI would probably have enhanced the verbalisation of ambivalence in the consultation. Although analysis suggested 'theoretical data saturation', the sample was small and other studies with a wider range of patient types and selection are required to investigate the generalisability of the findings to preventive consultations in general. Given the concept of grounded theory and its validation as used by Strauss and Corbin [33] and referring to the constructivist position by Charmaz [34] the analysis of ambivalence and its theoretical contents was derived from the empirical material and informed by the researcher's theoretical background and interpretive understanding of the meaning of the interviews. [35] The analytical concepts and categories were, furthermore, found to be consistent with the patients' lives and statements, even though some of the quotes may present ideal answers. None of the patients had previously participated in a preventive consultation about CVD, but according to their number of risk factors and their average age over 50 years, they had probably been exposed to opportunistic preventive messages from their normal consultations, which is a limitation of the study.

### The non-verbalisation of ambivalence in consultations

The frequent experience of ambivalence during the consultation makes it plausible that a preventive consultation induces ambivalence related to potential lifestyle changes among patients at increased risk of CVD. However, the patients found that GPs did not communicate systematically and purposefully about it. This could be so for several reasons. From the GP's perspective, the result could be attributed to a lack of knowledge about the frequent existence of ambivalence, a need for communicative education in handling of ambivalence or intentionally moving away from such issues in the consultation, for instance due to lack of time. Furthermore, an introduction to MI through written guidelines is inadequate to implement the communication strategy in preventive consultations in general practice. These challenges in relation to using MI with everyday practice are important potential causes of the non-systematic use of MI, evidenced by the relative lack of implementation of MI in this study (as judged by reports from these patients). From the patients' perspective, the results may reflect their difficulties in expressing ambivalence in the consultation or unconsciousness of their ambivalence before and during the consultation, which is underlined by the fact that the patients expressed that they were much more conscious of their ambivalence in the weeks after the consultation.

### The typology and informant characteristics

By analysing the ambivalence typology in the light of the informant characteristics we were not able to identify consistent relations between specific person characteristics and ambivalence subtypes. All patients experienced the different kind of ambivalences even though they had different age, gender and educational attainments.

### The typology and the naming of the ambivalence subtypes

The naming of each ambivalence subtype was made on the basis of the constructivist grounded theory approach [34] and the analytical round dance [35], where the interpreting part of the analysis is open for inspiring theoretical aspects, different grounded theory methodological strategies, the researcher and participants' constructions and interpretations of the consultation and the study objectives. Other suitable labels for the ambivalence subtypes may have been found when approaching another analytical frame or strategy in data collection, preparation and analysis.

### The Typology and the theoretical background

Our analysis brought forward five different ambivalence sub-types inhibiting patients' progress and preparedness to pursue lifestyle changes. These inhibitory influences are consistent with theories on the moderating effect of ambivalence in attitude-behaviour relationships, [29,30] information processing and change of attitude [2] and in particular, with the Theory of Reasoned Action (TRA) and of the Theory of Planned Behaviour (TPB) [36,37]. The TRA proposes that behaviour is predicted by intention to engage in that behaviour, which, in turn, is predicted by attitude towards that behaviour (a function of the perceived consequences of participation and a personal evaluation of those consequences) and the perceived social norm (a function of the perceived expectations to participate and the motivation to comply with those expectations). The TPB, a development of the TRA, was designed to expand the model to predict and explain behaviour that is not completely under the individual's volitional control. According to the TPB, whether someone intends to behave in a certain way depends on the extent to which that person perceives him or herself to be in control over a given behaviour, in addition to the attitudinal and social norm components included in the TRA [36,37].

At present, the non-verbalisation of ambivalence suggests that key behavioural determinants were not addressed enough in consultations, hence fundamentally undermining attempts to reduce risk behaviour. Furthermore, the sub-types can be matched to different TPB components. The perception ambivalence matches to perceived consequences, information ambivalence to personal evaluation of consequences, treatment and demand ambivalence to the perceived social norm and, finally, priority ambivalence to perceived behavioural control [37,38]. These sub-types may hence identify specific areas that can and should be addressed in consultations with the theoretical expectation and empirical backing [38] that this will enable patients to attempt and achieve lifestyle change more effectively than is currently the case.

Similarly, the different ambivalences can contribute to MI [7] and the trans-theoretical (stages of change) model of Prochaska and DiClemente [39]. The former is based on exploring ambivalence and behaviour change through the perceived importance and perceived confidence to change behaviour [7]. The latter consists of six different stages a person goes through in his/her lifestyle changing process from the pre-contemplation stage to the maintenance stage. The different ambivalence sub-types can be understood as an instance of mapping to different stages of MI – perception, information and treatment map to importance, and priority and demand map to confidence. In turn, they map to the earlier and later stages in the trans-theoretical model, respectively. These ambivalence sub-types hence represent specific areas that can be explored further in discussions between patients and GPs to promote lifestyle changes. However, what counts in the end, is not the GPs ability to have sophisticated discussions about ambivalence subtypes, but to improve the health profile of their patients through verbalisation of their ambivalence and increased awareness of its complexity and importance in relation to patients' motivation for lifestyle change. This can be attempted, based on the understanding gained from this study, but requires evaluation about its effectiveness for both patients and doctors.

This study did not aim to create new communication techniques, but rather to enhance existing evidence-based counselling methods in clinical practice and build on a theoretical basis for motivating and maintaining preventive health behaviour among patients at increased risk of CVD. It expands our knowledge about the central meaning of ambivalence in relation to lifestyle change and provides new insights into its complexity in the daily clinic. However, the results do not suggest that the GP should look for all the described ambivalence subtypes in the consultation to help the patient, but that the GPs and other health professionals are aware of the ambivalence phenomenon and its complexity in their motivational work with their patients. Our findings furthermore suggest that patients' ambivalence interacts with their reflections on lifestyle change in a concurrent process of managing conflicting thoughts and feelings in the consultation. This result is interesting from a cognitive psychological management perspective, because a cognitive-behavioural approach to this process of interaction aiming to change unhealthy, automated patterns of thoughts and feelings could prove instrumental in helping patients manage lifestyle change. It remains important to evaluate whether MI or other alternative cognitive strategies, enhanced by this understanding of ambivalence, could be clinically more effective in the communication about health determinants in the preventive consultations.

## Conclusion

Ambivalence and its subtypes were the core motivational aspects related to potential lifestyle changes in the interviews, even if they were not verbalised during the consultations. Ambivalence and its sub-types emerged from our data collection, preparation and analysis of these interviews following preventive consultations and seemed to interact with the patients' reflections on lifestyle change in a concurrent and iterative process. This study underlines the importance of using evidenced based motivational approaches as MI, because it aims to explore and resolve ambivalence as a central motivational aspect in relation to lifestyle change.

### Practice implications and future research

Future research and clinical work could explore why GPs do not talk about ambivalence in the consultations and what the consequences would be if they or other health professionals did so. Furthermore to investigate whether GPs and other health professionals' interventions to help patients verbalise their ambivalence will aid MI or other cognitive approaches thereby ensuring that patients' needs and concerns are addressed and that preventive consultations become even more effective. Finally other studies with a wider range of patient types and selection are required to investigate the generalisability of the results.

## Competing interests

The authors declare that they have no competing interests.

## Authors' contributions

DK, MBR, BC and TL are responsible for the design of the study and had the original idea. DK carried out the whole study, was responsible for the analysis and interpretation of results together with MBR and BC, wrote the first draft of the paper and made substantive intellectual contributions to it and will act as guarantor of the paper. MBR, MBC, BC, TL and AE have been involved in the data interpretation and drafting the manuscript or revising it critically for important intellectual content. All authors read and approved the final version of manuscript.

## Pre-publication history

The pre-publication history for this paper can be accessed here:


